# Neutrophil-to-Lymphocyte and Platelet-to-Lymphocyte Ratios as Predictors of Dysphagia Severity and Quality of Life in Nasopharyngeal Cancer Patients after Intensity Modulated Radiotherapy (IMRT)

**DOI:** 10.3390/jcm13164821

**Published:** 2024-08-15

**Authors:** Salvatore Cocuzza, Federica Maria Parisi, Corrado Spatola, Ignazio La Mantia, Jerome Rene Lechien, Carlos Chiesa-Estomba, Salvatore Ferlito, Gianluca Albanese, Mario Lentini, Miguel Mayo-Yanez, Nicolas Fakhry, Madalina La Rocca, Antonino Maniaci

**Affiliations:** 1 Department Scienze Mediche, Chirurgiche e Tecnologie Avanzate “G.F. Ingrassia”, Università di Catania, 95125 Catania, Italy; s.cocuzza@unict.it (S.C.); c.spatola@unict.it (C.S.); igolama@gmail.com (I.L.M.); ferlito@unict.it (S.F.); pasqualegianlucalbanese@gmail.com (G.A.); madalina.larocca@gmail.com (M.L.R.); 2 U.O. Radioterapia Oncologica, A.O.U. Policlinico “G. Rodolico-San Marco” Catania, Via Santa Sofia 78, 95123 Catania, Italy; 3 Head and Neck Study Group, Young-Otolaryngologists of the International Federations of Oto-Rhino-Laryngological Societies (YO-IFOS), 75000 Paris, France; jerome.lechien@umons.ac.be; 4 Department of Anatomy and Experimental Oncology, Mons School of Medicine, UMONS Research Institute for Health Sciences and Technology, University of Mons (UMons), 7022 Mons, Belgium; 5 Laboratory of Phonetics, Faculty of Psychology, Research Institute for Language Sciences and Technology, University of Mons (UMons), 7022 Mons, Belgium; 6 Department of Otorhinolaryngology and Head and Neck Surgery, CHU de Lille, Hôpital Claude Huriez, 59000 Lille, France; 7 Laryngopharyngeal Reflux Study Group, Young-Otolaryngologists of the International Federations of Oto-Rhino-Laryngological Societies (YO-IFOS), 13005 Paris, France; 8 Department of Otorhinolaryngology-Head and Neck Surgery, Hospital Universitario Donostia, 20014 San Sebastian, Spain; chiesaestomba86@gmail.com; 9 ASP Ragusa—Hospital Giovanni Paolo II, 97100 Ragusa, Italy; 10 Department of Otorhinolaryngology—Head and Neck Surgery, Complexo Hospitalario Universitario A Coruña (CHUAC), 15006 A Coruña, Spain; 11 Assistance Publique des Hôpitaux de Marseille, ORL et Chirurgie Cervico-Faciale, Hôpital de la Conception, 13005 Marseille, France; 12 Department of Medicine and Surgery, University of Enna “Kore”, 94100 Enna, Italy

**Keywords:** nasopharyngeal carcinoma, intensity modulated radiotherapy (IMRT), dysphagia, neutrophil-to-lymphocyte ratio (NLR), platelet-to-lymphocyte ratio (PLR)

## Abstract

**Background**: Patients treated with definitive radiotherapy for nasopharyngeal carcinoma (NPC) develop severe dysphagia, affecting their quality of life. Traditional prognosis biomarkers are insufficient, leading to a search for new predictors like neutrophil-to-lymphocyte ratio (NLR) and platelet-to-lymphocyte ratio (PLR). **Methods**: We retrospectively enrolled 44 NPC patients who underwent definitive radiotherapy between 2010 and 2018. EQUATOR and STROBE network guidelines were adopted. Pre-treatment evaluations were conducted, and post-treatment oropharyngeal dysphagia was assessed using the Sydney Swallow Questionnaire (SSQ) and FEES, then assigning a Dysphagia Outcome and Severity Scale (DOSS) level. Patients were divided based on NLR and PLR cut-offs, comparing subjective dysphagia (SSQ) scores and DOSS results at baseline and after a 5-year follow-up. Multiple linear regression was used for analysis. **Results**: At baseline, the mean NLR was 2.52 ± 1.10, and the PLR was 208.40 ± 94.35. Multivariate analysis indicated NLR and PLR as significant predictors of DOSS outcomes (*p* < 0.001). **Conclusions**: Baseline inflammation markers, such as NLR and PLR, may be used to predict dysphagia severity in NPC patients undergoing definitive radiotherapy. These markers could help identify patients at higher risk for severe dysphagia and implement tailored therapeutic and rehabilitative strategies to improve their quality of life. Further studies with larger cohorts are needed to confirm these findings and explore additional prognostic factors for dysphagia outcomes in NPC patients.

## 1. Introduction

Nasopharyngeal carcinoma (NPC) is a relatively uncommon epithelial carcinoma arising from the nasopharyngeal mucosal lining [1]. The geographical global distribution of nasopharyngeal carcinoma is unbalanced; >70% of new cases are in East and Southeast Asia [2]. Host genetics, EBV infection is perhaps nasopharyngeal carcinoma’s most common causal agent [3]. Other potential risk factors include active and passive tobacco smoking and consumption of preserved foods [4,5]. While the eighth edition of the TNM classification remains the most widely utilized prognostic tool for nasopharyngeal carcinoma (NPC), it is important to consider several additional variables for an accurate long-term prognosis [6]. Recent research has focused on functional imaging, plasma biomarkers, and molecular tumour characteristics [7,8,9]. Certainly, inflammation markers play an important role in the prognostic definition of cancer, influencing the tumour’s progression and survival [10]. Novel biomarkers have been proposed in the literature, such as the neutrophil-lymphocyte ratio (NLR), platelet count, lymphocyte-to-monocyte ratio (LMR), and the CRP/albumin ratio [11,12]. A recent meta-analysis by Takenaka et al. found that NLR greater than the cut-off value was associated with poor overall survival (HR 1.51, 95% CI 1.27–1.78), disease-specific survival (HR 1.44, 95% CI 1.22–1.71), progression-free survival (HR 1.53, 95% CI 1.22–1.90), and distant metastasis-free survival (HR 1.83, 95% CI 1.14–2.95) [13]. Although (Radiotherapy) RT for NPC has led to increased survival rates, it frequently causes burdensome symptoms, such as mucositis, dysphagia, taste disorders, and xerostomia [14]. Dysphagia is one of the most prevalent and challenging late adverse effects of radiotherapy in patients with NPC [15]. Ultimately, having easily accessible and actionable biomarkers would be beneficial to quickly implement dietary modifications and personalized nutrition plans for NPC patients. An interesting retrospective study showed that in patients with IMRT, along with N3 stage and concurrent chemotherapy, total cholesterol, LDL-C, and albumin levels were predictors for dysphagia [16]. Additionally, these markers could aid in educating patients about the signs and symptoms of aspiration, ensuring they report such incidents to their healthcare providers without delay. This study was designed to explore the relationship between readily accessible inflammatory biomarkers and the severity of dysphagia in patients with NPC undergoing radiotherapy.

This study was designed to explore the relationship between readily accessible inflammatory biomarkers and the severity of dysphagia in patients with NPC undergoing radiotherapy. Additionally, we aimed to investigate other potential predictors of dysphagia outcomes, including demographic and clinical factors, to provide a comprehensive understanding of the variables influencing dysphagia in this patient population.

## 2. Materials and Methods

All patients affected by squamous NPC treated with definitive RT or Radiochemotherapy (RCT) between January 2010 and January 2018 were retrospectively reviewed using information from a single tertiary institute. An average 5-year follow-up was required for each patient. We retrieved studies describing the design, conduct, and reporting of randomized clinical studies from the EQUATOR network (https://www.equator-network.org/) (accessed on 1 May 2024). We then selected and adhered to the Strengthening the Reporting of Observational Studies in Epidemiology (STROBE) guidelines [17]. All participants included were assessed at baseline and 5-year follow-up. The study complied with the Helsinki Declaration and policies approved by the local board of ethical committee (IRB.19912021/PO). All subjects with the following features were excluded from the study:

- Patients with a previously diagnosed dysphagia;
- Previous pneumonia;
- Previous cancers at any other sites or prior radiotherapy or chemotherapy;
- Clinical conditions might affect the NLR or PLR, such as inflammatory, autoimmune, acute, or chronic infectious disease, haematological or neurological disorders, a history of corticosteroid therapy, or chronic renal insufficiency. Clinical history, physical examination, and laboratory tests were performed at baseline. All the tumours were retrospectively staged according to the TNM classification (eighth Edition). As protocol, all participants included were subjected to fiberoptic swallowing evaluation (FEES) and subjective questionnaires for dysphagia at baseline and post IMRT 5-year follow-up. Informed consent was obtained from all the subjects involved in the study.

During the 5-year follow-up period, patients were seen at the oncologic evaluation every 3 months for the first year and then every 6 months afterward. All examinations were repeated at each of these follow-up visits.

### 2.1. IMRT Protocol

The study database was sampled to include patients treated with definitive IMRT for stage I-II squamous cell carcinoma of the nasopharynx. An image-guided radiotherapy and adaptive re-planning paradigm was used to administer IMRT to patients enrolled in this study. Adaptive rescheduling was performed for all study patients at least once based on daily CT-on-rails images. Target volumes and local therapy allocation were based on initial staging, performing radiation as a single mode for T1–T2 tumours. Gross and margin disease was given a dose of 66 Gy in 30 fractions for T1 disease and 72 Gy in 40–42 fractions with a concurrent boost fractionation scheme for patients with T2 tumours. All irradiation schedules were planned for 6 weeks of therapy.

### 2.2. Swallowing Assessment

Oropharyngeal dysphagia was established by irregular swallowing physiology estimated by a clinical, bedside screening test and consequently confirmed by FEES assessment in all the participants. Oropharyngeal dysphagia was screened by a trained speech-language pathologist (SLP) through a clinical evaluation that included a 3 oz water test, which assessed the patient’s features before and after water intake. SPL administered 10 mL of water from a glass; coughing, regurgitation, laryngeal movement, and a drop in oxygen saturation were assessed [18]. A score ≤ 8 on the 3 oz water test was used as a cut-off to indicate oropharyngeal dysphagia. Thus, Bedside examination was performed via the Sydney Swallowing Questionnaire (SSQ) [19]. The SSQ evaluates swallowing difficulties, especially in neurogenic, oropharyngeal dysphagia patients. The SSQ consists of 17 well-structured questions for assessing and quantifying patient-reported difficulties in swallowing function. The questions cover the symptoms related to combinations of variables like the anatomic region, type of dysfunction, and the consistency of swallowed bolus. SSQ was employed as a patient-reported outcome measure. The individual question scores are calculated on a 100 mm visual analogue scale, with a higher score indicating a more severe swallowing impairment; these scores were examined as a continuous variable.

Consequently, two different Otolaryngologists experienced in the swallowing evaluation assessed dysphagia severity through flexible endoscopic swallowing evaluation (FEES) (C.S., A.M.). The Dysphagia Outcome Severity Scale (DOSS) was used [20]. DOSS was considered our main outcome measure for dysphagia severity because it may be used to assess the severity of dysphagia objectively and has therapeutic significance. In our research, we employed DOSS levels as an ordinal variable, where lower levels correspond to more severe dysphagia. Each procedure was video-recorded to facilitate the interpretation (Olympus-Olympus Corporation-Japan). The DOSS is a 7-point scale developed to rate the dysphagia severity during video-fluoroscopy and suggest diet recommendations; however, several literature studies also validated the DOSS reliability for FEES [21,22]. According to DOSS outcomes, patients were subsequently divided into different swallowing classes, from Stage 7, considered normal alimentation, to Stage 1, which identifies patients with severe dysphagia and no possible oral food intake (Table 1).

### 2.3. FEES Protocol

**Figure 1:**
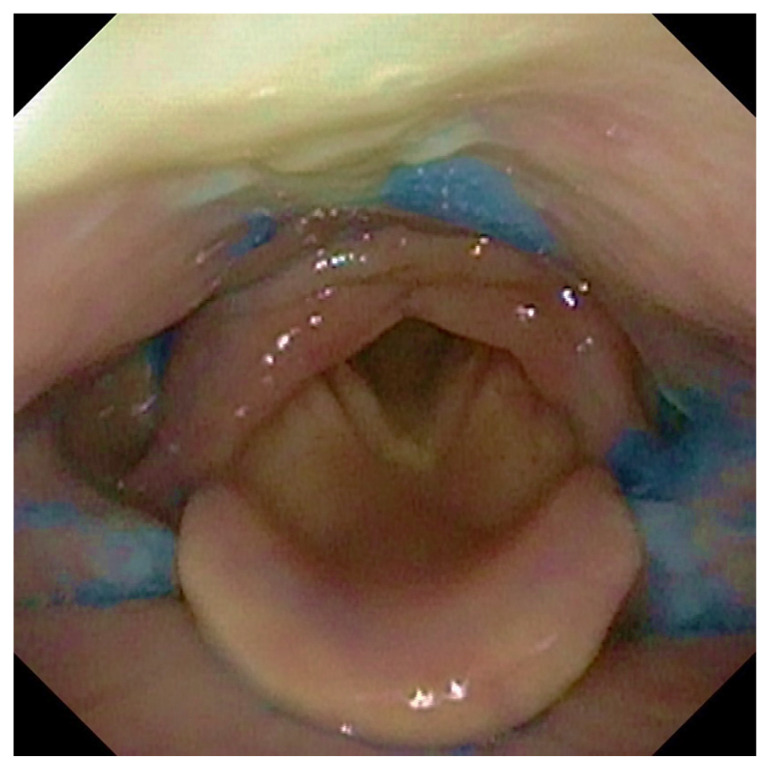
The figure shows the presence of post-swallowing residue of water coloured with methylene blue at the level of the retro arytenoid space and the bilaterally in pyriform sinuses.

Participants were offered three tests of thin liquid and three of thick liquid. In each test, we administered 10 ccs of water with thin liquid and 10 ccs with thick liquid of aqua gel (Nestlè Nutricia Nutilis^®^, Danone SA, Milan, Italy) dyed with Methylene Blue for food (Figure 1). The hard and soft solid textures were evaluated without signs of dysphagia with thin or dense liquids. We rated solids ingestion with a banana as a soft food and a cracker as a hard one. The same food consistencies were administered in all patients analyzed.

### 2.4. Statistical Analysis and Data Comparison

Data analysis was performed using IBM SPSS Statistics for Windows, IBM Corp., Released 2017, Version 25.0, Armonk, NY, USA: IBM Corp. Descriptive statistics were reported on average ± standard deviation or proportion. The T-test for paired samples was used to determine the difference between observations for normally distributed data. The Mann–Whitney U test was performed to analyze group differences for continuous skewed data. So, we divided patients into two groups according to the cut-off defined in the literature for NLR and PLR [23]. We compared subjective scores related to perceived dysphagia and the results obtained through the DOSS to the baseline and at the end of the 5-year follow. The ANOVA test assessed the differences between the groups in NLR, PLR, and DOSS levels. 

Moreover, the different dependent variables that could influence the swallowing function of the patients enrolled, such as age, sex, and marital status, were also evaluated. Multivariate analysis was used to estimate the relationship between a dependent and an independent variable. In a multiple linear regression analysis, we used NLR, PLR, and additional potential predictors, such as clinical and demographic characteristics, as independent variables and DOSS or SSQ levels as the dependent variable. To minimize the risk of spurious findings when testing multiple hypotheses, we employed the Bonferroni correction (αnew = αoriginal/n). This method adjusted each test’s significance level to maintain an acceptable overall error rate. Specifically, in multivariate analysis, 8 variables were tested, and we divided the standard alpha level of 0.05 by 28, resulting in a Bonferroni-corrected alpha of *p* = 0.0017 for each hypothesis test.

## 3. Results

Among the 83 initial enrolled subjects, 44 (20 female and 24 male) eligible patients were included. Demographic features are summarized in Table 2. Low education status was reported in 33 (75%) subjects. Only 4 (9.09%) participants had completed middle school, and 7 (15.9%) had a higher education. Marital status was reported in 27 subjects (61.7%) (*p* = 0.033). Any differences in weight loss were observed among participants, with a majority experiencing a slight weight loss of >3 kg (40.9%; *p* = 0.283). Regarding the clinical tumour extension, a slight difference was observed among cT1 and cT2 tumours (54.55% vs. 45.45%; *p* = 0.393). Variability was found for inflammatory biomarkers among participants. The NLR scored 2.52 ± 1.11, while the PLR had a value of 208.41 ± 94.35, demonstrating a broad range of inflammatory states (Table 2).

### 3.1. Dysphagia Outcomes, Demographics, and Blood Biomarkers

**Figure 2:**
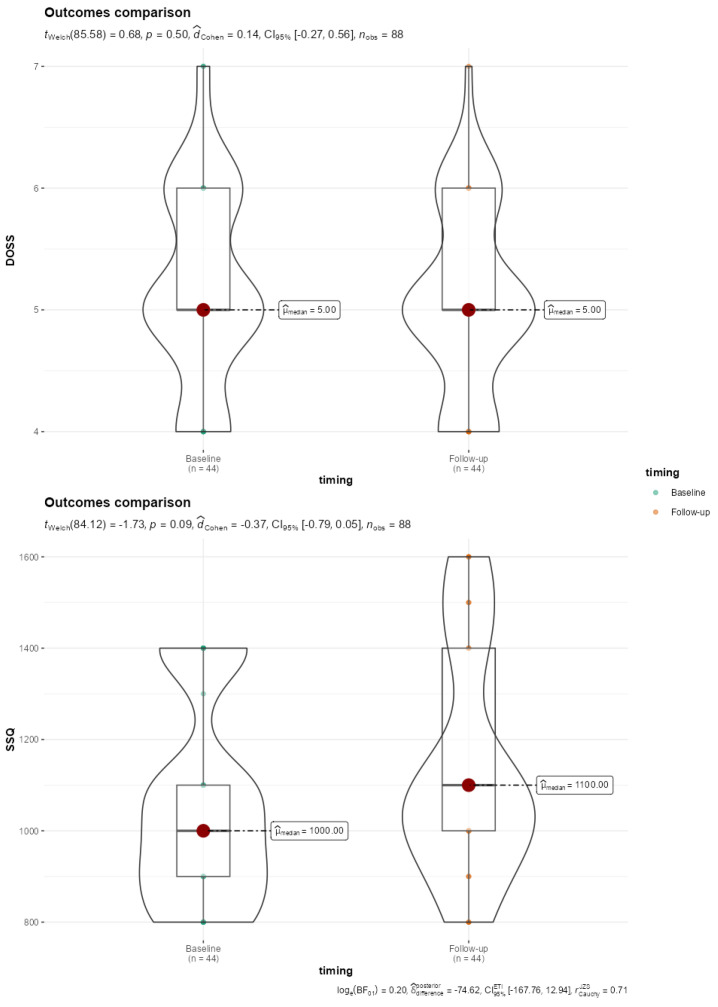
Violin-Box plot reporting comparison of DOSS and SSQ baseline and 5-year follow-up outcomes. SSQ, Sydney Score questionnaire; DOSS, Dysphagia Outcomes Severity Scale.

Only two patients showed no difficulty swallowing (level 7); 20 patients had mild dysphagia (5 levels), while 9 showed mild to moderate dysphagia (level 4). Although worse Dysphagia Outcome and Severity Scale (DOSS) scores were found at follow-up compared to baseline, the difference did not reach statistical significance (Figure 2). Regarding the subjective dysphagia evaluation, we obtained an average SSQ score of 1136.36 ± 242.23, indicating varied swallowing difficulties among participants. No statistical significance was found with baseline data comparison (*p* = 0.088) (Figure 2). 

**Figure 3:**
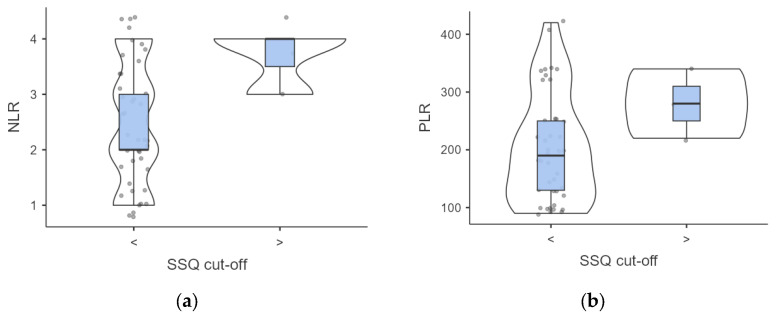
(**a**,**b**) Intergroup analysis of inflammatory biomarkers outcomes according to SSQ score. Abbreviations: SSQ, Sydney Swallow Questionnaire; NLR, neutrophil to lymphocyte ratio; PLR. Platelet to Lymphocyte ratio.

A complex association between dysphagia severity and inflammatory markers was found. Participants with elevated SSQ scores demonstrated higher but not significant baseline NLR (3.66 ± 0.47 vs. 2.43 ± 1.08, *p* = 0.063) and PLR (280 ± 49.98 vs. 203.17 ± 93.58; *p* = 0.188) values compared to those with normal SSQ scores (Figure 3).

**Figure 4:**
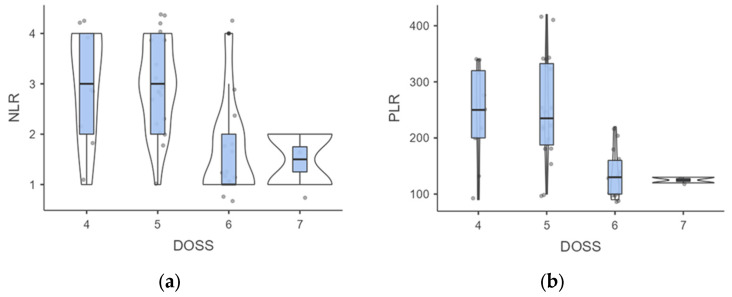
(**a**,**b**). Intergroup analysis of inflammatory biomarkers outcomes according to DOSS levels. Abbreviations: NLR, neutrophil to lymphocyte ratio; PLR. Platelet to Lymphocyte ratio; DOSS, Dysphagia Outcomes Severity Scale.

When examining DOSS levels at follow-up, we found that level 4 individuals had higher but not significant NLR than those at level 7 (3 ± 1.05 vs. 1.5 ± 0.71; *p* = 0.109), probably due to the low sample of the subgroups (Figure 4). 

A similar trend was observed for PLR outcomes (241.11 ± 84.78 vs. 125 ± 7.07; *p* = 0.113). However, a significant difference was noted in the comparison between level 4 vs. level 6, where both NLR (3 ± 1.05 vs. 1.69 ± 0.95; *p* = 0.007) and PLR (241.11 ± 84.78 vs. 133.85 ± 43.88; *p* = 0.001) outcomes were significantly different. When stratified by DOSS outcomes, the Kruskal-Wallis test confirmed significant differences in the NLR (H = 13.63; *p* = 0.003) and PLR scores (H = 15.66; *p* = 0.001). Mild to moderate dysphagia affected 40.9% of patients exhibiting a low NLR and 90.9% of patients with elevated NLR levels (*p* < 0.001). In addition, the incidence of mild to moderate dysphagia appeared to be substantially higher in individuals with a higher PLR compared to just those with a low PLR (91.3% vs. 38.1%; *p* < 0.001).

### 3.2. Predictive Variables of Dysphagia Outcomes

Among demographic features, unmarried individuals presented a significantly higher risk of experiencing mild to moderate dysphagia compared to their married counterparts, with an odds ratio of 1.702 (95% CI: 1.137–2.546) (Table 3). 

In addition, the prevalence of mild to moderate dysphagia was highest among individuals with a low instructional level at 82.8%, compared to 6.9% and 10.3% for medium and high levels, respectively; however, no significance was found (*p* = 0.248). A significant association between dysphagia and weight loss categories was found (*p* = 0.009). While mild to moderate dysphagia was observed in 44.4% of individuals with <3 kg weight loss increased to 66.7% among those with moderate weight loss (3 to 5 kg) and reached 100% in cases with more than 5 kg weight loss. In addition, at multivariate analysis predicting mild to moderate dysphagia, marital status, and weight loss, NLR and PLR emerged as significant predictors (Table 4). SSQ scores were nonsignificant after the Bonferroni test when predicting with DOSS (F = 4.044, *p* = 0.051). In addition, Marital status did not find a significant association with dysphagia expressed by DOSS (F = 6.819, *p* = 0.012) or SSQ (F = 5.523, *p* = 0.024); instead, baseline weight loss (F = 11.182, *p* = 0.002), NLR (F = 19.298, *p* < 0.001) and PLR (F = 21.741, *p* < 0.001) presented a strong correlation only for DOSS outcomes even after Bonferroni correction. 

## 4. Discussion

Our study delved into the complex interplay between dysphagia and long-term patient outcomes, focusing on identifying predictors that could influence swallowing performance and improve patient care strategies [5,6,24]. In a three-year post-radiotherapy follow-up observational study, Szczesniak et al. did not find a correlation between dysphagia severity and the tumour’s stage, patient age, or sex [25]. Our study confirmed that demographic factors exhibit a complex relationship with the dysphagia severity. We found no significant differences in dysphagia severity based on gender or age. Our findings examined the importance of social support systems and nutritional status in patient outcomes. However, we did not find a significant correlation between marital status and dysphagia severity or DOSS outcomes (F = 6.819, *p* = 0.012) or subjective findings as SSQ scores (F = 5.523, *p* = 0.024). Although our findings demonstrated a higher risk for the severity of dysphagia and marital status, this finding should be interpreted cautiously due to the small sample size, repeated comparisons, and possible confounding factors. Larger, more carefully monitored investigations are required to confirm this connection and investigate the underlying mechanisms. This suggests that the emotional support, practical assistance, and motivation provided by a partner are not crucial factors in managing the challenges of dysphagia more effectively [26,27]. Some articles have discussed the predictive value of weight loss to highlight the need for early nutritional interventions. A recent study involving 55 patients with locally advanced head and neck (H&N) cancer found a significant correlation between aspiration pneumonia, dysphagia, and weight loss [27]. In our study, weight loss presented a compelling dichotomy as a predictor of dysphagia outcomes. While it was strongly correlated with dysphagia severity at DOSS (F = 11.182, *p* = 0.002), its correlation with the SSQ variable (F = 1.210, *p* = 0.278) was not statistically significant.

In contrast, education level appeared to have a more complex relationship with dysphagia outcomes in our study. Patients with a low level of education reported more cases of mild to moderate dysphagia. The educational level could be indirectly related to patient outcomes, potentially through factors such as health literacy and the ability to understand and manage treatment and its side effects. Note, however, that educational level did not reach statistical significance as a predictor in multivariate analysis for DOSS (F = 2.742; *p* = 0.105) and SSQ (F = 0.939; *p* = 0.338) scores. In addition, a particularly noteworthy aspect of our findings is the intricate relationship between dysphagia severity and inflammatory markers. Ku et al. recently highlighted the role of inflammation in altering the outcomes of older patients with H&N cancer, suggesting that it should be considered a significant risk factor for assessment [28]. Among inflammatory biomarkers that are easy to obtain and interpret, NLR and PLR have recently been studied and are useful in predicting treatment response and outcomes in neoplasms of the H&N district [23]. 

The study’s pivotal finding is the robust association between inflammatory biomarkers and the severity of dysphagia. Patients with elevated NLR and PLR levels were significantly more likely to experience mild to moderate dysphagia. Probably, the baseline inflammation might contribute to tissue damage or enhance the body’s sensitivity to the adverse effects of radiotherapy, leading to increased dysphagia severity. We observed that participants with higher SSQ scores had elevated NLR and PLR baseline levels. However, the differences in NLR (*p* = 0.063) and PLR (*p* = 0.188) did not achieve statistical significance, possibly due to the small sample size. When stratifying by DOSS levels, individuals at level 4 exhibited higher, though not statistically significant, NLR (*p* = 0.109) and PLR (*p* = 0.113) values than those at level 7. This could be attributed to our study’s small subgroups, which may limit the power to detect statistically significant differences.

In contrast, the comparison between DOSS levels 4 and 6 revealed significant differences, with level 4 displaying significantly higher NLR (*p* = 0.007) and PLR (*p* = 0.001) values, suggesting a correlation between inflammation and mild to moderate dysphagia. These findings were further supported by the Kruskal-Wallis test, which indicated significant differences in inflammatory markers across different dysphagia outcomes, with both NLR (H = 13.63; *p* = 0.003) and PLR (H = 15.66; *p* = 0.001) scores showing significant intergroup variation. Interestingly, baseline NLR and PLR emerged as significant predictors of DOSS performance and SSQ scores among the variables examined. 

### Study Limitations

Although promising findings were revealed, it is crucial to acknowledge the study’s limitations. The small sample size may limit the generalizability to other populations or settings. For several reasons, our study’s conclusions were mostly based on DOSS results. First, compared to subjective patient-reported measures, we felt that DOSS delivers an objective, clinician-assessed assessment of dysphagia severity and hence offers a more trustworthy indicator of swallowing function. Our findings are more applicable because DOSS is widely accepted in clinical practice and has undergone extensive validation.

Lastly, DOSS enables a standardized assessment more easily comparable among patients and studies, whereas SSQ offers insightful information about patients’ perspectives. To give a complete picture of dysphagia in our patient cohort, we have included SSQ results and emphasized the significance of patient-reported outcomes. The possible confounding influence of age on the observed relationship between dysphagia severity and married status must be taken into account. It may sound paradoxical, but our research revealed that a higher risk of dysphagia was linked to younger age. This finding, however, could be explained by several variables, including variations in the features of the tumor, variables connected to the treatment, physiological variables, or behavioral variables. Younger patients, for instance, can have more aggressive tumor subtypes when they first arrive or might have more rigorous treatment plans, which could increase their risk of developing dysphagia.

Additionally, the onset and progression of radiation-induced dysphagia may be influenced by aging-related changes in muscle composition and swallowing function. Additionally, younger individuals likely handle side effects from treatment differently, which could affect their ability to swallow. Extensive investigation is required to examine these plausible rationales and clarify the intricate correlation between age and dysphagia severity in individuals with nasopharyngeal cancer. Furthermore, it is important to recognize the limitations of this study, including the relatively small sample size and the possibility of residual confounding, and to proceed with caution when interpreting the findings. When analyzing the relationship between factors such as Instructional Level, Weight Loss, and dysphagia scores, there was not enough sample size to fully capture all potential combinations of the variables. This restriction might have impacted the analysis’s statistical power and capacity to identify meaningful correlations between the relevant variables. Future research should strive to enlist a larger sample size to overcome this limitation and guarantee that all possible combinations of the variables are fairly represented in the data. Additionally, the retrospective design means that causality cannot be inferred, and there may be unmeasured confounding factors. 

It is crucial to recognize that this study’s limited sample size resulted in missing data, making it impossible to analyze some variables, including instruction level and weight reduction. This restriction might have affected the careful evaluation of the relationships between the factors under investigation. The results should be interpreted cautiously, and more studies with larger cohorts are required to validate and build upon these discoveries. These constraints may affect the broader applicability of our conclusions, underlining the necessity for further studies with a stronger study design. 

## 5. Conclusions

Inflammation biomarkers show promise for predicting dysphagia severity in nasopharyngeal carcinoma patients. Conversely, demographic features such as gender, weight loss, and educational level may present varied influences. Future efforts should focus on confirming these findings in larger populations, understanding the underlying mechanisms, and developing interventions to improve patient outcomes.

## Figures and Tables

**Table 1:** Dysphagia Outcome and Severity Scale (DOSS) is a 7-point scale developed to rate the functional severity of dysphagia systematically. Abbreviations: DOSS, Dysphagia Outcome, and Severity Scale; P.O., Per OS.

Level 7	Level 6	Level 5	
Normal in all situations Normal diet. No strategies or extra time is needed.	Within functional limits/modified independence Normal diet, functional swallow. The patient may have a mild oral or pharyngeal delay, retention or trace epiglottal undercoating but independently and spontaneously compensates/clears. May need extra time for meal Have no aspiration or penetration across consistencies.	Mild dysphagia: Distant supervision; may need one diet consistency restricted. May exhibit one or more of the following: aspiration of thin liquids only but with a strong reflexive cough to clear completely; airway penetration midway to cords with one or more consistency or to cords with one consistency but clears spontaneously; retention in the pharynx that is cleared spontaneously; mild oral dysphagia with reduced mastication and/or oral retention that is cleared spontaneously.	
**Level 4**	**Level 3**	**Level 2**	**Level 1**
Mild–moderate dysphagia: Intermittent supervision/cueing, one or two consistencies restricted. May exhibit one or more of the following: retention in pharynx cleared with a cue, retention in the oral cavity that is cleared with a cue, aspiration with one consistency, with weak or no reflexive cough or airway penetration to the level of the vocal cords with cough with two consistencies or airway penetration to the level of the vocal cords without cought with one consistency	Moderate dysphagia: Total assistance, supervision, or strategies, two or more diet consistencies restricted, may exhibit one or more of the following: moderate retention in the pharynx, cleared with a cue, moderate retention in the oral cavity, cleared with a cue, airway penetration to the level of the vocal cords without cough with two or more consistencies or aspiration with two consistencies, with weak or no reflexive cough or aspiration with one consistency, no cough and airway penetration to cords with one, no cough.	Moderately severe dysphagia: Maximum assistance or use of strategies with partial P.O. only (tolerates at least one consistency safely with total use of strategies). May exhibit one or more of the following: severe retention in the pharynx, unable to clear or needs multiple cues; Severe oral stage bolus loss or retention, unable to clear or needs multiple cues; Aspiration with two or more consistencies; no reflexive cough, weak volitional cough or aspiration with one or more consistency, no cough and airway penetration to cords with one or more consistency, no cough.	Severe dysphagia: Unable to tolerate any P.O. safely. May exhibit one or more of the following: severe retention in the pharynx, inability to clear, severe oral stage bolus loss or retention, inability to clear, silent aspiration with two or more consistencies, nonfunctional volitional cough, or inability to achieve swallow.

**Table 2:** Main demographics features at preoperative assessment. Abbreviations: CHT, chemotherapy; SSQ, Sydney swallowing questionnaire; NLR, neutrophil to lymphocyte ratio; PLR. Platelet to Lymphocyte ratio; DOSS, Dysphagia Outcomes Severity Scale.

Variable	n = 44	Mean ± sd/n%
Gender	Male	24 (54.55%)
	Female	20 (45.45%)
Age		55.97 ± 9.63 yo
Marital status	Yes	27 (61.7%)
	No	17 (38.3%)
Instruction level	Low	33 (75%)
	Middle	4 (9.09%)
	High	7 (15.9%)
Clinical tumor staging	cT1	24 (54.55%)
	cT2	20 (45.45%)
Clinical Nodal staging	cN0	22 (50%)
	cN1	11 (25%)
	cN2	11 (25%)
CHT	yes	42 (95.45%)
	no	2 (4.55%)
Loss of weight	<3 kg	18 (40.9%)
	>3 kg <5 g	15 (34.09%)
	>5 g	11 (25%)
DOSS	Level 4	9 (20.45%)
	Level 5	20 (45.45%)
	Level 6	13 (29.54%)
	Level 7	2 (4.54%)
SSQ		1052.2 ± 208.33
NLR		1.95 ± 0.64
PLR		207.54 ± 94.93

**Table 3:** Risk estimation for both DOSS and SSQ outcomes. Abbreviations: SSQ, Sydney Swallowing Questionnaire; NLR, neutrophil to lymphocyte ratio; PLR; Platelet to Lymphocyte ratio; DOSS, Dysphagia Outcomes Severity Scale; OR, Odd Ratio. The limited sample size resulted in empty cells within the contingency table for Instructional Level and Weight Loss variables, precluding the calculation of a reliable odds ratio estimate.

	DOSS			SSQ		
	CI 95%			CI 95%		
Variable	OR	Inferior	Superior	OR	Inferior	Superior
Age	0.622	0.176	2.202	0.432	0.036	5.145
Gender	1.077	0.308	3.762	1.727	0.145	20.578
Marital Status	1.702	1.137	2.546	0.824	0.661	1.026
Instructional level	-	-	-	-	-	-
Weight Loss	-	-	-	-	-	-
SSQ/DOSS	0.634	0.503	0.8	1.115	0.986	1.262
NLR	14.444	2.682	77.796	1.158	0.981	1.367
PLR	17.063	3.127	93.106	1.15	0.982	1.347

**Table 4:** Multivariate analysis for predictive dependent variables of mild to moderate dysphagia. Abbreviations: SSQ, Sydney Swallowing Questionnaire; NLR, neutrophil to lymphocyte ratio; PLR; Platelet to Lymphocyte ratio; DOSS, Dysphagia Outcomes Severity Scale. To account for the multiple comparisons involving 8 variables, each hypothesis was tested using a Bonferroni-corrected alpha level of *p* = 0.00625. This corrected alpha was determined by dividing the standard alpha level of 0.05 by the number of variables (8).

	DOSS		SSQ	
Dependent Variable	F	*p*-Value	F	*p*-Value
Age	0.121	0.729	0.139	0.711
Gender	0.013	0.91	0.183	0.671
Marital status	6.819	0.012	5.523	0.024
Instruction level	2.742	0.105	0.939	0.338
Weight loss	11.182	0.002	1.210	0.278
SSQ/DOSS	4.044	0.051	7.853	0.008
NLR	19.298	<0.001	3.629	0.064
PLR	21.741	<0.001	1.892	0.176

## Data Availability

The original contributions presented in the study are included in the article, further inquiries can be directed to the corresponding author.
